# Co-expression network of neural-differentiation genes shows specific pattern in schizophrenia

**DOI:** 10.1186/s12920-015-0098-9

**Published:** 2015-05-16

**Authors:** Mariana Maschietto, Ana C Tahira, Renato Puga, Leandro Lima, Daniel Mariani, Bruna da Silveira Paulsen, Paulo Belmonte-de-Abreu, Henrique Vieira, Ana CV Krepischi, Dirce M Carraro, Joana A Palha, Stevens Rehen, Helena Brentani

**Affiliations:** LIM23 (Medical Investigation Laboratory 23), University of Sao Paulo Medical School (USP), São Paulo, SP Brazil; Institute of Psychiatry-University of Sao Paulo, Medical School (FMUSP), São Paulo, SP Brazil; Hospital Israelita Albert Einstein, São Paulo, Brazil; Post-graduation Program Institute of Mathematics and Statistics, University of Sao Paulo, São Paulo, SP Brazil; Institute of Biomedical Sciences, Federal University of Rio de Janeiro, Rio de Janeiro, Brazil; Department of Psychiatry, Federal University of Rio Grande do Sul, Porto Alegre, Brazil; Institute of Biosciences, University of São Paulo, São Paulo, SP Brazil; International Research Center-AC Camargo Cancer Center, São Paulo, Brazil; Life and Health Sciences Research Institute (ICVS), School of Health Sciences, University of Minho, Braga, Portugal; ICVS/3B’s-PT Government Associate Laboratory, Braga, Guimarães Portugal; D’Or Institute for Research and Education (IDOR), Rio de Janeiro, Brazil; Department of Psychiatry, University of Sao Paulo, Medical School (FMUSP), Rua Dr Ovídio Pires de Campos,785-CEP 05403-010, São Paulo, SP Caixa Postal n 3671 Brazil; National Institute of Developmental Psychiatry for Children and Adolescents, CNPq, São Paulo, SP Brazil

**Keywords:** Schizophrenia, Gene network, Neuronal differentiation, Module analyses, Oxidative stress

## Abstract

**Background:**

Schizophrenia is a neurodevelopmental disorder with genetic and environmental factors contributing to its pathogenesis, although the mechanism is unknown due to the difficulties in accessing diseased tissue during human neurodevelopment. The aim of this study was to find neuronal differentiation genes disrupted in schizophrenia and to evaluate those genes in post-mortem brain tissues from schizophrenia cases and controls.

**Methods:**

We analyzed differentially expressed genes (DEG), copy number variation (CNV) and differential methylation in human induced pluripotent stem cells (hiPSC) derived from fibroblasts from one control and one schizophrenia patient and further differentiated into neuron (NPC). Expression of the DEG were analyzed with microarrays of post-mortem brain tissue (frontal cortex) cohort of 29 schizophrenia cases and 30 controls. A Weighted Gene Co-expression Network Analysis (WGCNA) using the DEG was used to detect clusters of co-expressed genes that werenon-conserved between adult cases and controls brain samples.

**Results:**

We identified methylation alterations potentially involved with neuronal differentiation in schizophrenia, which displayed an over-representation of genes related to chromatin remodeling complex (adjP = 0.04). We found 228 DEG associated with neuronal differentiation. These genes were involved with metabolic processes, signal transduction, nervous system development, regulation of neurogenesis and neuronal differentiation. Between adult brain samples from cases and controls there were 233 DEG, with only four genes overlapping with the 228 DEG, probably because we compared single cell to tissue bulks and more importantly, the cells were at different stages of development. The comparison of the co-expressed network of the 228 genes in adult brain samples between cases and controls revealed a less conserved module enriched for genes associated with oxidative stress and negative regulation of cell differentiation.

**Conclusion:**

This study supports the relevance of using cellular approaches to dissect molecular aspects of neurogenesis with impact in the schizophrenic brain. We showed that, although generated by different approaches, both sets of DEG associated to schizophrenia were involved with neocortical development. The results add to the hypothesis that critical metabolic changes may be occurring during early neurodevelopment influencing faulty development of the brain and potentially contributing to further vulnerability to the illness.

**Electronic supplementary material:**

The online version of this article (doi:10.1186/s12920-015-0098-9) contains supplementary material, which is available to authorized users.

## Background

Schizophrenia is a severe and debilitating neuropsychiatric disorder with a worldwide prevalence of approximately 1 % and an estimated heritability of 80–85 % [1]. Multiple factors contribute to its pathogenesis, including genetic and environmental factors [2–4], which are involved in the disturbance of the neuronal network formation and brain function [5].

Although the genetic component involvement is clear, the precise molecular and cellular defects that precedes illness onset and the sequence of mechanisms that initiate or attenuate disease progression, particularly in neurons, are still unknown. In addition to findings from animal models [6–8], post-mortem human brain studies in schizophrenia revealed significant alterations in genes with high expression in multiple cortical and hippocampal regions [9–13]. Data from prefrontal cortex obtained post-mortem revealed 28 modules of co-expressed genes associated to oxidative phosphorylation, energy production and metabolism, post-translation modifications, neurogenesis and neuronal differentiation. Additionally, these modules showed a robust effect of age suggesting that genes related to nervous system development process and neuronal differentiation are deregulated in schizophrenia [14]. A co-expression module analysis from patients with schizophrenia (SCZP) and controls of seven studies showed that the top-modules of each study displayed a significant enrichment of neuronal markers as well as genes related to the electron transport chain and oxidative phosphorylation biological processes [15]. Eventually, these alterations, through the analyses of genes and proteins, can be found in the peripheral blood suggesting that they are potential molecular markers for diagnosis or follow-up [16–19].

Promising approaches involve the study of tissue from the olfactory system by nose biopsies [20] and the development of human induced pluripotent cells (hiPSC) techniques. The later demonstrated that functional primarily glutamatergic neurons differentiated from hiPSC were able to fire action potentials after eight weeks in culture, showing the possibility of studying schizophrenia through this technique [21]. SCZP-hiPSC-derived neurons display diminished neuronal connectivity with reduced neuritis and changes in Wnt and cAMP signaling pathways when compared to controls [22]. Additionally, RNA sequencing analysis of hiPSCs-derived neurons highlighted the importance of long non-coding RNAs in neurogenesis [23].

In a previous study, we showed that neural progenitor cells (NPCs), derived from hiPSCs from skin fibroblasts of a schizophrenic patient had a 2-fold increase in extramitochondrial oxygen consumption compared to normal controls, and correspondingly elevated levels of reactive oxygen species (ROS), which was reduced to control levels by addition of valproic acid [24]. Mitochondrial dysfunction was also associated with impaired differentiation of neurons into both mature dopaminergic and glutamatergic phenotypes in schizophrenia-derived hiPSCs [25].

Although genes involved with ROS and other molecular mechanisms in hiPSC and NPC have been studied in patients with schizophrenia [26], the ones specifically disrupted in neuronal differentiation were not assessed directly in adult brain samples. The aim of this study was to disclose gene expression changes during neuronal differentiation disrupted in schizophrenia and to expand these findings to the brain tissue in adults. To achieve that, genes identified as involved with neuronal differentiation exclusively in schizophrenia were analyzed by co-expression network in post-mortem brain samples derived from adult patients and controls. Then, we identified the biological processes associated with a less conserved module between cases and controls brain samples.

## Methods

### Tissue collection

In short, biopsies of primary human fibroblasts from a 48 year-old woman with DSM-IV-TR schizophrenia defined as treatment-resistant and under clozapine treatment (SZCP) were collected in parallel with a control subject (CON) that negative for any major lifetime DSM-IV-TR diagnosis. Fibroblasts underwent primary culture procedures to generate hiPSCs and were subsequently differentiated into neurons (NPC). Details were previously described [24].

Total RNA was isolated from three plates of cell culture (with molecular confirmation for the cell type that was being studied [24]) using Trizol (TRI Reagent, Sigma-Aldrich, St Louis, MO, USA) according to the manufacturer’s instructions. RNA quantity and integrity were assessed by spectrophotometry (NanoDrop ND-2000 UV–Vis Spectrophotometer, NanoDrop Technologies) and microfluidics-based electrophoresis (Agilent 2100 Bioanalyzer; Agilent Technologies, Santa Clara, California), respectively, and obtained OD of ~2.0 and RIN > 9. Genomic DNA was extracted using standard proteinase K digestion followed by phenol/chloroform extraction. DNA quantity and integrity were assessed by spectrophotometry (NanoDrop ND-2000 UV Spectrophotometer, NanoDrop Technologies) and agarosis gel 0.8 %.

The study protocol was approved by the ethics committee of the Hospital de Clínicas de Porto Alegre, Porto Alegre (RS) with all subjects providing written informed consent co-signed by close relatives (Register 09-108). The study was performed in accordance with the Declaration of Helsinki.

### Comparative genomic hybridization based on microarray (array-CGH)

We performed comparative genomic hybridization based on microarrays (array-CGH) in a commercial whole-genome 180 K platform containing 180,000 oligonucleotide probes (design 30864, Agilent Technologies, Palo Alto, California) using as reference DNA a commercially available human pool of healthy individuals (Promega). Purification, cohybridization of labelled test and reference DNA samples and washing were carried out according to the manufacturer’s instructions. Scanned images were processed using the Feature Extraction 10.7.3.1 software (Agilent Technologies, Palo Alto, California).

### Methylation experiments

Bisulfite-conversion of 500 ng of DNA was performed according to the manufacturer’s recommendations (EZ DNA Methylation Gold Kit, Zymo Research, Irvine, California) for the Illumina Infinium Assay. The incubation profile was 16 cycles at 95 °C for 30 s, 50 °C for 60 min and a final holding step at 4 °C for 10 min. In total, 10 μl of bisulfite-converted DNA were used for hybridization on Infinium HumanMethylation 450 BeadChip with 485,557 probes, following the Illumina Infinium HD Methylation protocol (Illumina, Inc, San Diego, California). All arrays passed Illumina standard quality control metrics and were included in this study.

### Microarray experiments

For mRNA expression, samples were prepared according to the Agilent mRNA Microarray System protocol using 250 ng of total RNA for amplification and labelled using the Agilent Low RNA Input Fluorescent Linear Amplification Kit. Samples were hybridized in the Agilent Whole Human Genome Microarray Kit, 4 × 44 K-G4112F in the Agilent SureHyb chambers (Agilent Technologies, Palo Alto, California) for 20 h at 55 °C, and the platforms were washed with the manufacturer’s washing buffers. Controls were used in all labelling reactions. Scanned images of the arrays were processed using Feature Extraction 10.7.3.1 software (Agilent Technologies, Palo Alto, California) with default parameters. Quality control of the probes (i.e. low intensity, saturation, controls, etc.) selected 30,635 probes corresponding to 22,186 transcripts for the analyses. From those, 14,491 probes correspond to unique genes and 7695 were not associates with an ENTREZID. Four genes had more than one ENTREZID with the same Official Symbol and were excluded from the analyses.

Frontal cortical samples from a previous study were used for further analyses, all presenting good quality total and amplified RNA [27]. Postmortem brain tissue was donated by The Stanley Medical Research Institute's brain collection. Details about ethics regarding these samples are available at the Neuropathology Consortium (http://www.stanleyresearch.org). Comparing patients with schizophrenia and controls, there were no significant differences concerning age, brain weight or PMI, with only pH showing a significant difference (*p* = 0.01). In short, 30 controls subjects (CTS) and 29 patients with schizophrenia (SZP) from the Stanley Foundation were hybridized in the Agilent 4x44K human oligonucleotide microarray (Agilent 4112 F, Agilent Technologies, Palo Alto, California) following manufacturer procedures. For analysis of replicate spots, the average intensity after background correction was calculated, followed by normalization using locally weighted linear regression (LOWESS) with α = 0.2 within slides using the R software version 2.11.1 (R Development Core Team, 2010).

Data were deposited in NCBI Gene Expression Omnibus and are accessible through GEO Series accession number GSE62191 and GSE62105.

### Data analyses

DNA copy number variations (CNVs) were identified with Nexus software 7.0 (Biodiscovery, Hawthorne, CA, USA) using the FASST2 segmentation algorithm. To be considered a CNV, the criteria were: a minimum of 3 consecutive affected probes (effective resolution of ~50 Kb for CNV calling), a significance threshold set at 5.0e^−8^, and threshold log_2_ ratio Cy3/Cy5 of 0.4 for gain and −0.4 for loss. Data from sex chromosomes were excluded. Gene annotation was performed according to the GRCh37/hg19using the University of California Santa Cruz Genome Browser (http://genome.ucsc.edu/) and the Database of Genomic Variants (DGV; May 2014 update).

Due to the low number of samples, we simplified the methylation analyses. CpGs with β-values ≤0.2 were considered hypo-methylated and with β-values ≥0.8 were considered hyper-methylated, which were used to construct Venn-diagrams to compare the data in fibroblasts, hiPSC and NPC.

For gene expression analyses, it was applied HT-self [28]. Self–self experiments were performed by labelling the hIPSC from SZCP with either Cy3 or Cy5 dyes and hybridizing simultaneously on the same microarray slide to determine the intensity dependent cut-offs. These cut-offs were applied to the non-self–self experiment that was performed in triplicate (SCZP × CON). To identify differentially expressed genes, three criteria were applied: a. each gene must be represented by at least two probes; b. more than 50 % of the probes representing that gene presented reliable signal; c. there was at least 80 % of signal agreement among probes. To avoid technical variability, only genes that reached the three criteria in all replicates were selected.

To investigate if genes identified in this study could have a role during foetal development of the prefrontal cortex (dorsolateral prefrontal cortex, ventrolateral prefrontal cortex, anterior (rostral) cingulate (medial prefrontal) cortex), they were searched in the BrainSpan database (Allan Brain Atlas) [29]. Genes with at least 1 RPKM in more than 75 % of the samples or those who had an expression interquartile range >0.5 were considered for the analyses. Genes were assigned as foetal or non-foetal according to the development stage of the sample. It was considered differentially expressed those genes with fold-change >2.

The Wilcoxon Rank Sum Test was applied using TMEV software package [30] to identify differentially expressed genes between 29 SZP and 30 CTS considering those with *p* < 0.01 as significantly differentially expressed. As pH was significantly different in the brains between cases and controls, we accessed the DEG which expression was possibly being affected by pH. Hence, a multiple regression analysis was applied for all expressed genes in the microarray platform with a further comparison to 1000 permutated datasets. We observed that the difference of pH in the brain affects around 20 % of expressed genes (F, *p* < 0.05) of the whole array. These genes were compared to the DEGs between SZP and CTS.

The DEG sets found by both analyses were compared to the findings of a published study that defined 17 modules comprised of co-expressed genes associated with human cortical development. Briefly, to define human brain development modules, RNA-sequencing data from neocortical regions of the BrainSpan were analyzed through co-expression networks using the R package Weighted Gene Co-expression Network Analysis (WGCNA) [31]. The identified modules were characterized using GO-Elite [32] for controlling the network-wide false discovery rate. It was significant those pathways that had >10 genes at Z-summary >2 and FDR < 0.01 [31]. To estimate the enrichment of the DEG sets among the 17 modules, the Modular Single-set Enrichment Test (MSET) was applied with 10,000 permutations [33]. For that, we calculated the probability of the DEG being significantly represented in each module compared to 10,000 simulated sets of genes generated randomly from the microarray background. For each DEG and module, the *p*-value was calculated based on the number of simulated randomized sets that contains equal or higher number of genes belonging to the DEG in that module. Scatter smooth plots were construct only for the modules that had DEG over-representation (*p*-value ≤0.05) using the module eigengene values identified by the study [31].

In this study, the preservation module function from the WGCNA package in R were used to build unsigned co-expression networks to characterize features of co-expressed gene modules [34–36]. A pairwise correlation matrix was computed for all gene pairs across all samples, and an adjacency matrix was calculated by raising the correlation matrix to the power of 7. This threshold was chosen based on the scale-free topology criterion, which is the smallest threshold that resulted in a scale-free R^2^ fit about 0.8, and used for all networks: schizophrenia samples only and controls samples only. For each pair of genes, a robust measure of network interconnectedness (topological overlap measure) was calculated based on the adjacency matrix. The topological overlap based dissimilarity was then used as input for average linkage hierarchical clustering. Finally, modules were defined as branches of the resulting clustering tree. To cut the branches, we used the hybrid dynamic tree-cutting in order to have robustly defined modules. The minimum module size was set to 20 genes with the deepSplit parameter set to 2. Each module was summarized by the first principal component of the scaled (standardized) module expression profiles (ME). Hub genes were assigned as having high intramodular connectivity kME >0.75. To discriminate changes in modules comparing SZP and CTS, we performed a module Preservation analysis that identified which modules were preserved in both datasets. For that, a Z-summary was calculated to exclude the possibility of randomness in preservation based on 1000 permutations, and indicating if a module is strongly (Z-summary >10), moderately (2 < Zsummary <10) or not preserved (Z-summary <2). For visualization, the resulting modules were plotted using Cytoscape [37].

For DEG, functional and disease enrichment analyses were performed in WebGestalt [38] using the whole genome as background with a further analysis implemented in DAVID (database http://david.abcc.ncifcrf.gov/). It was considered those biological processes and pathways that comprised more than 10 genes and FDR < 0.05.

## Results

### Characterization of molecular alterations potentially interfering with gene expression

To refine the identification of genes regulated during neuronal differentiation, copy number alterations were characterized by array comparative genomic hybridization (CGH). Most of the detected copy number variations (CNVs) were present in all cell types (fibroblasts, hiPSCs and NPCs), which precludes their characterization as an acquired copy number alteration related to cell differentiation. There was no CNVs found exclusively in NPC from SCZP. Nevertheless, one large gain at 20q11.21 (1.2 MB) was acquired by the hiPSC and maintained in the NPC from the SCZP. The region contains 32 genes (Additional file 1: Figure S1). There were no CNVs found exclusively in NPC from SCZP.

To explore methylation alterations potentially involved in neuronal differentiation in schizophrenia, we compared NPCs, hiPSC and fibroblast from SCZP and CON, selecting CpG sites differentially methylated exclusively between hiPSCs and NPCs in SCZP and CON. A Venn-diagram identified 51 (33 genes) and 5 (3 genes) CpG sites as hypo- or hyperrmethylated, exclusively in SCZP, respectively (Additional file 1: Figure S2, Table S1). These genes were over-represented in specific locations of the genome, 1p36, 10q22, 12q22 and 14q24 (adjusted *p*-value, adjP < 0.05). We also found overrepresentation of chromatin remodeling complex (adjP = 0.04), represented by *DPF3*, *SMARCA2* and *RERE. DPF3b* interacts with the histones H3 and H4 in an acetylation-sensitive manner to regulate transcription [39]. *SMARCA2* is a member of the SWI/SNF family of proteins with helicase and ATPase activities and thought to activate transcription altering chromatin structure in specific sites [40]. *RERE* is a nuclear receptor co-repressor that binds to the histone methyltransferase G9a orchestrating molecular events that lead to a stable methylation of histone H3-lysine 9 [41].

### Characterization of expression patterns in SCZP

Gene expression profiles from hiPSC (SCZP or CON) were compared to the corresponding groups of differentiated cells (i.e. NPCs) to identify differences during neuronal differentiation. We considered that the analyses in CON-derived cells identified DEG during neuronal differentiation; there were 1658 up-regulated (range from 0.50 to 6.76) and 1698 down-regulated (ranging from −0.43 to −8.49) genes. Comparison of the SCZP-derived cells revealed 752 up-regulated (range from 0.56 to 3.4) and 818 down-regulated (ranging from −0.54 to −4.97) genes. Subsequently, to exclude genes associated with normal neuronal differentiation, up- and down-regulated genes in CON and SCZP were compared to select those that were exclusive for SCZP. The analysis revealed 228 genes (151 up- and 77 down-regulated) altered only during neuronal-differentiation of the SCZP cells (Additional file 1: Table S2, Figure S3), from which ten were described by studies in schizophrenia. Most of them were related to nervous system development and/or plasticity: *NDEL1, ATF4, AKT1, MDGA1, PDE4B, FEZ1, TF3, ADRBK2, CACNA1C, ADRA2A* (Additional file 1: Table S3). In agreement with our data, 10 genes (*ATF4, CACNA1C, CPE, CTSF, EPHA2, GPR155, HOXB9, MYBL1, NLGN1,* and *PMAIP1*) were identified by an independent study that used a similar design (hiPSC x NPC in schizophrenia cases) [26].

Using expression data from the prefrontal cortex of Brain Span (Allen Brain Atlas), expression of the 228 gene set were compared between 16 foetal and 22 non-foetal samples. Nine genes were not present in Allen Atlas and 35 did not pass quality control (see material and methods). From the remaining 184 genes, 73 (40 %) were differentially expressed and therefore considered important for brain development.

To explore the functional significance of the genes from neurodevelopment associated with schizophrenia, we performed unbiased Gene Ontology term enrichment and pathway analyses of the 228 genes. The most noteworthy biological processes that were overrepresented were metabolic processes, signal transduction, nervous system development, regulation of neurogenesis and neuronal differentiation. Among the cellular pathways, we found over-representation of genes related to MAPK signaling pathway, pathways in cancer and metabolic pathways (Table 1). Finally, it was interesting to note that genes involved with cancer, infectious, metabolic and brain disorders (including stress, bipolar disorder and schizophrenia) were overrepresented diseases (Table 2).

**Table 1:** Signaling cellular pathways over-represented among the 228 gene set

Pathway	Statistical test	Genes
MAPK signalling pathway	C = 268;O = 13;E = 1.38;R = 9.42; *p* = 1.47e09;adjP = 1.50E-07	*PDGFA, ATF4, AKT1, CACNA1C,*
		*DUSP6, CACNA1E, CACNA1H, NLK,*
		*CHUK, MAP3K5, NTF3, FOS, PPM1V*
Pathways in cancer	C = 326;O = 13;E = 1.68;R = 7.75; *p* = 1.80e08;adjP = 7.74E-07	*PDGFA, RARA, AKT1, ITGA2, E2F3,*
		*FH, SMO, CHUK, FZD5, FOS, BID,*
		*SPI1, HDAC2*
Metabolic pathways	C = 1130;O = 21;E = 5.82;R = 3.61; *p* = 4.84e07;adjP = 1.39E-05	*IMPA1, TRDMT1, HSD17B8, DPM1,*
		*MGAT4B, CPOX, FH, SPHK1, DCT,*
		*GAL3ST1, PNMT, PIK3C2A, ASNS, PC, MTHFS, PPAP2B, CDO1, HAAO,*
		*ATP6V1C1, ALDH1A1, AGL*

Criteria: at least 10 genes represented in the pathway and adjpvalue ≤0.05 (hypergeometric *t* test with p value adjusted by multiple test adjustment, Benjamini and Hochberg [69])

*C* number of reference genes in the category number of genes in the gene set and *O* in the observed category, *E* expected number in the category, *R* ratio of enrichment, *p p* value from hypergeometric test, *adjP* value adjusted by the multiple test adjustment (adjP)

**Table 2:** Diseases over-represented among the 228 gene set

Disease	Statistical test	Genes
Cancer or viral infections	C = 951;O = 25;E = 4.90;R = 5.11; *p* = 3.35e-11;adjP = 8.71e-10	*RARA, WIF1, AKT1, LGALS3, RB1CC1, E2F3, FH, SPHK1,*
		*DCT, FOS, BID, PDGFA, CD24, S100A7, EPHA2, PMAIP1,*
		*SMO, RHOBTB2, CTNND1, SPI1, ETV1, ALDH1A1, CEACAM1, HDAC2, MYB*
Neoplasms	C = 854;O = 22;E = 4.40;R = 5.00; *p* = 7.90e-10;adjP = 1.03e-08	*RARA, WIF1, AKT1, LGALS3, E2F3, FH, DCT, PDGFA, CD24,*
		*S100A7, EPHA2, PMAIP1, FLT1, SMO, RHOBTB2, CTNND1, SSTR1, SPI1, ETV1, ALDH1A1, CEACAM1, MYB*
Drug interaction with drug	C = 349;O = 13;E = 1.80;R = 7.24; *p* = 4.00e-08;adjP = 3.47e-07	*CEBPB, PPIB, KPNA4, RARA, AKT1, PMAIP1, ITGA2, ACTA1, PACS1, STX4, FOS, BID, HDAC2*
HIV	C = 755;O = 18;E = 3.89;R = 4.63; *p* = 9.30e-08;adjP = 6.04e-07	*ADARB1, PPIB, KHDRBS1, MGAT4B, FURIN, PSMD7, PACS1,*
		*FOS, EED, PIK3C2A, CEBPB, PSMB10, KPNA4, MANEA, DDX6, ACTA1, GTF2H1, HLA-DOA*
Bipolar disorder	C = 344;O = 12;E = 1.77;R = 6.78; *p* = 2.71e-07;adjP = 1.41e-06	*IMPA1, ATF4, DUSP6, CACNA1C, MDGA1, CTSF, CIT, PDE4B, FEZ1, NLGN1, NTF3, ADRBK2*
Infection	C = 516;O = 14;E = 2.66;R = 5.27; *p* = 5.72e-07;adjP = 2.48e-06	*MIF, PSMB10, PPIB, KPNA4, TBK1, MICB, FURIN, PACS1, NKRF, IL13RA1, TICAM1, HLA-DOA, CEACAM1, PIK3C2A*
Metabolic diseases	C = 612;O = 15;E = 3.15;R = 4.76; *p* = 8.02e-07;adjP = 2.61e-06	*PC, PEX6, JAZF1, CST3, CPOX, TOP3A, APOA2, NEFH, ADIPOR2, PYGL, LIPA, AGL, APOA1, BCL11A, ZMPSTE24*
Leukemia	C = 452;O = 13;E = 2.33;R = 5.59; *p* = 7.65e-07;adjP = 2.61e-06	*RCBTB1, RARA, ASNS, AKT1, HUWE1, PMAIP1, PRDM16, DOK1, CASC5, BID, SPI1, BCL11A, MYB*
Stress	C = 464;O = 13;E = 2.39;R = 5.44; *p* = 1.02e-06;adjP = 2.95e-06	*ASNS, ATF4, MTF1, AKT1, PMAIP1, MICB, ACTA1, SGK1, CHUK, MAP3K5, FOS, PRDX4, RPL11*
Mental disorders	C = 564;O = 14;E = 2.90;R = 4.82; *p* = 1.62e-06;adjP = 3.79e-06	*NDEL1, CACNA1C, MDGA1, ASPM, BACE1, CST3, PDE4B, NEFH, FEZ1, NLGN1, NTF3, ADRBK2, CDK5R1, ADRA2A*
Adhesion	C = 647;O = 15;E = 3.33;R = 4.50; *p* = 1.59e-06;adjP = 3.79e-06	*TEK, CD24, AKT1, LGALS3, EPHA2, RB1CC1, MDGA1, ITGA2,*
		*ACTA1, CADM3, PTPN12, NLGN1, CTNND1, CEACAM1, CLDN11*
Neoplastic processes	C = 411;O = 12;E = 2.12;R = 5.67; *p* = 1.75e-06;adjP = 3.79e-06	*WASF3, CD24, WIF1, AKT1, LGALS3, EPHA2, FLT1, SPHK1, CTNND1, ETV1, ALDH1A1, CEACAM1*
Death	C = 343;O = 11;E = 1.77;R = 6.23; *p* = 1.94e-06;adjP = 3.88e-06	*RBM20, ATF4, AKT1, PMAIP1, SPHK1, HRK, BCLAF1, CHUK, MAP3K5, BID, CDK5R1*
Leukemia, myeloid	C = 279;O = 10;E = 1.44;R = 6.96; *p* = 2.14e-06;adjP = 3.97e-06	*RARA, HUWE1, PMAIP1, PRDM16, DOK1, CUX1, HRK, BID, SPI1, MYB*
Neoplasm	C = 298;O = 10;E = 1.53;R = 6.52; *p* = 3.84e-06;adjP = 6.24e-06	*WASF3, CD24, AKT1, LGALS3, EPHA2, FURIN, CUX1, CTNND1, ETV1, CEACAM1*
Invasiveness		
Nervous system diseases	C = 694;O = 15;E = 3.57;R = 4.20; *p* = 3.73e-06;adjP = 6.24e-06	*ATXN7, USH1G, PC, PEX6, ASPM, BACE1, DUX4, CST3, SOX3, NEFH, CACNA1H, FOXG1, GIGYF2, XK, CDK5R1*
Breast	C = 377;O = 11;E = 1.94;R = 5.67; *p* = 4.79e-06;adjP = 7.33e-06	*S100A7, CD24, AKT1, LGALS3, EPHA2, RB1CC1, FXYD3, RHOBTB2, ALDH1A1, HDAC2, MYB*
Neoplasms		
Brain diseases	C = 411;O = 11;E = 2.12;R = 5.20; *p* = 1.08e-05;adjP = 1.56e-05	*ATXN7, PC, PEX6, BACE1, CST3, NEFH, AQP1, CACNA1H, FOXG1, GIGYF2, CDK5R1*
Schizophrenia	C = 360;O = 10;E = 1.85;R = 5.40; *p* = 1.98e-05;adjP = 2.57e-05	*NDEL1, ATF4, AKT1, CACNA1C, MDGA1, PDE4B, FEZ1, NTF3, ADRBK2, ADRA2A*
Central nervous system diseases	C = 438;O = 11;E = 2.25;R = 4.88; *p* = 1.94e-05;adjP = 2.57e-05	*ATXN7, PC, PEX6, BACE1, CST3, NEFH, CACNA1H, FOXG1, GIGYF2, XK, CDK5R1*
Virus diseases	C = 488;O = 11;E = 2.51;R = 4.38; *p* = 5.16e-05;adjP = 6.39e-05	*CXCL5, PSMB10, KPNA4, TBK1, MICB, ACTA1, PSMD7, TICAM1, HLA-DOA, FOS, PIK3C2A*
Carcinoma	C = 522;O = 11;E = 2.69;R = 4.09; *p* = 9.37e-05;adjP = 0.0001	*S100A7, CD24, WIF1, AKT1, LGALS3, EPHA2, SMO, FH, CTNND1, ALDH1A1, CEACAM1*
Pathologic processes	C = 561;O = 10;E = 2.89;R = 3.46; *p* = 0.0007;adjP = 0.0008	*MIF, CXCL5, CD24, HTRA1, PRDM16, JAZF1, ITGA2, FLT1, ZMIZ1, LOXL1*
Syndrome	C = 654;O = 10;E = 3.37;R = 2.97; *p* = 0.0022;adjP = 0.0024	*WASF3, USH1G, PEX6, WASL, TOP3A, FH, FOXG1, LOXL1, XK, ZMPSTE24*
Genetic	C = 808;O = 10;E = 4.16;R = 2.40; *p* = 0.0095;adjP = 0.0099	*MIF, CACNA1C, HTRA1, JAZF1, ITGA2, PDE4B, ZMIZ1, LOXL1, GIGYF2, ADRA2A*
Predisposition to		
Disease		

Criteria: at least 10 genes represented in the pathway and adjpvalue ≤0.05 (hyper-geometric *t* test with p value adjusted by multiple test adjustment, Benjamini and Hochberg [69])

*C* number of reference genes in the category number of genes in the gene set and *O* in the observed category, *E* expected number in the category, *R* ratio of enrichment, *p p* value from hypergeometric test,. *adjP* value adjusted by the multiple test adjustment (adjP)

There were no overlap between the 228 DEG and genes from the 20q11.1 gain observed in the schizophrenic case. *BACE1* was the only gene that was also identified by the methylation analysis. This gene seems to regulate proteins with important roles in developmental process such as *NGR1* [42], which overexpression results in a schizophrenia-like behavior in a mouse model [43].

### Comparison of deregulated genes from individual cells and brain bulk samples

Comparison of gene expression profiling from 29 SZP and 30 CTS (brain bulks) revealed 233 differentially expressed transcripts (*p* < 0.01), of which 69 are unknown transcripts. We observed 127 (75 genes) up- and 106 (89 genes) down-regulated transcripts, none of them surviving the multiple correction. Furthermore, difference in pH of the brain between SZP and CTS is affecting 78 (33 %) of the differentially expressed transcripts (Additional file 1: Table S4).

To gain further information of the 228 and 233 DEG sets, they were mapped to the gene co-expression modules from human cortical development [31]. The enrichment analysis point out that each set of DEGs had over-represented genes but in different modules (M). While the set of 228 genes was enriched in M8, the set of 233 genes was enriched in M1, M5 and M15 (Additional file 1: Table S5). M8 and M15 were representative of genes preferentially expressed in “early” and “late” foetal period in human brain development, respectively (Additional file 1: Figure S4). This finding suggests that both datasets are related to neurodevelopment genes, but to different stages of the brain development.

To expand our findings from a cellular model of neuronal differentiation to postmortem samples from human brains, the 228 gene set, found exclusively in SCZP were compared to the 233 differentially expressed genes from adult brain samples. There were 194 genes out of the 228 represented in both platforms, from which four genes were common to both analyses: *AQP1*, *DUSP6*, *FOS* and *TRIM24*. Only *DUSP6* and *FOS* have been described in schizophrenia [44, 45]. None of the them were affected by the pH difference.

**Fig. 1:**
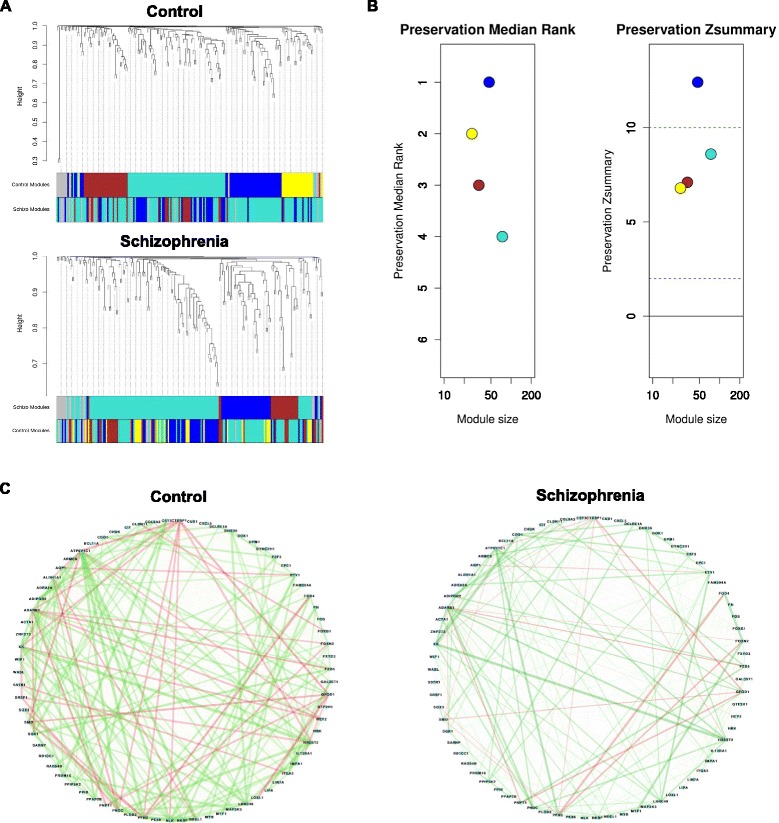
Module preservation analysis. **a** Dendrograms produced by average hierarchical clustering using topological overlapping matrix dissimilarity. Colours represent different modules. *Upper panel* shows modules in control subjects (CTS; *blue*, *brown*, *yellow* and *turquoise*) compared to patients with schizophrenia (*SZP*). Lower panel shows modules in patients with schizophrenia (*blue*, *brown* and *turquoise*) compared to modules in control subjects. **b** *Left panel* shows the median preservation rank (y-axis) in relation to module size (x-axis). Each circle represents a module labelled in different colours (*blue*, *turquoise*, *yellow* and *brown*). *Right panel* shows the Zsummary (y-axis) in function of module size. *Dashed lines* represent thresholds 2 and 10: ≥10: high preservation; 2 < Zsummary <10: moderate preservation; <2: low preservation. The panels show that the *blue module* is more preserved in control and patients whilst the *turquoise module* is less preserved. **c** Connectivity patterns (correlation network adjacencies) between genes from the *turquoise module* in controls (*control*) and patients (*schizophrenia*) showing a large loss of connectivity among genes in patient’s module compared to *control modules*. Line thickness represents the connectivity pattern and line colour reflects the absolute correlation: −1 (*negative*) to 1 (*positive*). This module is enriched in genes related to response to negative regulation of cell differentiation and oxidative stress

Further, to identify functional modules in patients and controls, a co-expression analyses of the 194 genes was performed in WGCNA. Modules for CTS and SZP were generated using the Scale-free Topology Criterion with power 7. Four modules were identified plus the grey module (Fig. 1a). Genes and biological processes overrepresented in each module are displayed in Tables 3 and 4, respectively. The best preserved module in SZP compared to CTS is the blue (blue, Z-summary >10, Fig. 1B) which had an overrepresentation of genes related to cell cycle.

**Table 3:** Genes preserved in the modules of the respective samples

Module	Genes
Blue	*AGL, ANAPC4, APOA1, ARMCX6, ASPM, ATF4, BCLAF1, BID, CAPRIN1, CENPJ, CLIP4, CPOX, CTNND1,*
	*EDNRA, EIF3A, EIF5, EPHA2, FAM133B, GMFB, GRB10, HLA-DOA, HSD17B8, ING5, KHDRBS1, KPNA4,*
	*LYPD6, LZTS1, METTL16, MOCS3, NLGN1, NPR1, NRL, ORMDL1, PC, PSMB10, PSTK, RANBP2, RINT1, SMG5, SPHK1, STX4, USH1G, WASF3, WDR44, ZMIZ1, ZNF426, ZNF99*
Brown	*CACNA1C, CACNA1E, CCAR1, CEACAM1, EED, EIF3J, FURIN, GJD3, HDAC2, HUWE1, ING3, LGALS3,*
	*LOC389634, MANEA, METAP2, MRPL19, MTHFS, NEK6, NOC3L, NUP107, PDK2, PMAIP1, PPM1B,*
	*PRDX4, PTPN12, PYGL, RCBTB1, SCAF8, SNTB1, TEK, TRIM24, USP1, ZMPSTE24*
Grey	*CCDC149, CEBPB, DDX6, FLT1, GIGYF2, KCNK15, KDELC2, RARA, RARS, SLC38A4, TBK1, TMEM208, TRDMT1, ZNF614*
Turquoise	*ACTA1, ADARB1, ADIPOR2, ADRA2A, ALDH1A1, AQP1, ARMC8, ATP6V1C1, BCL11A, CDO1, CHUK, CIT,*
	*CLDN11, COL9A3, CST3, CTDSP1, CUX1, CXCL5, DCLRE1A, DHX36, DOK1, DPM1, DYNC2H1, E2F3,*
	*EPC1, ETV1, FAM204A, FGD4, FH, FOS, FOXG1, FOXN2, FXYD3, FZD5, GAL3ST1, GFOD1, GTF2H1,*
	*HEY2, HRK, HS6ST2, IL13RA1, IMPA1, ITGA2, LIN7A, LIPA, LOXL1, LRRC49, MAP3K5, MTF1, MYB,*
	*NDEL1, NKRF, NLK, PEX6, PFN2, PLOD2, PNOC, PNPT1, PPAP2B, PPIB, PPIP5K2, PRDM16, RAD54B,*
	*RB1CC1, SARNP, SGK1, SMO, SOX3, SRSF1, SSTR1, WASL, WIF1, XK, ZNF273*
Yellow	*ADRBK2, AKT1, ASIC2, ASNS, CACNA1H, CADM3, CD24, CDK5R1, DUSP6, FBRS, GPR155, HNRNPH1, MDGA1, MGAT4B, MICB, MYBL1, PACS1, PDE4B, PDGFA, PNMT, PPP1R13B, RANGRF, RHOBTB2, RIPK4, SPRY4, TICAM1*

**Table 4:** Biological processes overrepresented in the modules

Module	GO ID	Ontology name	Ontology definition
Blue	GO:0010564	regulation of cell cycle process	Any process that modulates a cellular process that is involved in the progression of biochemical and morphological phases and events that occur in a cell during successive cell replication or nuclear replication events.
	GO:0000086	G2/M transition of mitotic cell cycle	Progression from G2 phase to M phase of the mitotic cell cycle. The molecular event responsible for this transition is the activation of the major cell cycle cyclin-dependent kinase (e.g. Cdc2 in S. pombe, CDC28 in S. cerevisiae, Cdk1 in human).
	GO:0051325	interphase	A cell cycle process comprising the steps by which a cell progresses through interphase, the stage of cell cycle between successive rounds of chromosome segregation. Canonically, interphase is the stage of the cell cycle during which the biochemical and physiologic functions of the cell are performed and replication of chromatin occurs.
	GO:0051329	interphase of mitotic cell cycle	Interphase occurring as part of the mitotic cell cycle. Canonically, interphase is the stage of the cell cycle during which the biochemical and physiologic functions of the cell are performed and replication of chromatin occurs. A mitotic cell cycle is one which canonically comprises four successive phases called G1, S, G2, and M and includes replication of the genome and the subsequent segregation of chromosomes into daughter cells.
	GO:0007346	regulation of mitotic cell cycle	Any process that modulates the rate or extent of progress through the mitotic cell cycle.
	GO:0000278	mitotic cell cycle	Progression through the phases of the mitotic cell cycle, the most common eukaryotic cell cycle, which canonically comprises four successive phases called G1, S, G2, and M and includes replication of the genome and the subsequent segregation of chromosomes into daughter cells. In some variant cell cycles nuclear replication or nuclear division may not be followed by cell division, or G1 and G2 phases may be absent.
	GO:0045184	establishment of protein localization	The directed movement of a protein to a specific location.
	GO:0016482	cytoplasmic transport	The directed movement of substances (such as macromolecules, small molecules, ions) into, out of, or within the cytoplasm of a cell.
Brown	GO:0006508	proteolysis	The hydrolysis of proteins into smaller polypeptides and/or amino acids by cleavage of their peptide bonds.
	GO:0030163	protein catabolic process	The chemical reactions and pathways resulting in the breakdown of a protein by the destruction of the native, active configuration, with or without the hydrolysis of peptide bonds.
	GO:0044257	cellular protein catabolic process	The chemical reactions and pathways resulting in the breakdown of a protein by individual cells.
	GO:0044267	cellular protein metabolic process	The chemical reactions and pathways involving a specific protein, rather than of proteins in general, occurring at the level of an individual cell. Includes cellular protein modification.
	GO:0042593	glucose homeostasis	Any process involved in the maintenance of an internal steady state of glucose within an organism or cell.
	GO:0019538	protein metabolic process	The chemical reactions and pathways involving a specific protein, rather than of proteins in general. Includes protein modification.
	GO:0006325	chromatin organization	Any process that results in the specification, formation or maintenance of the physical structure of eukaryotic chromatin.
	GO:0016568	chromatin modification	The alteration of DNA, protein, or sometimes RNA, in chromatin, which may result in changing the chromatin structure.
Grey	GO:0017148	negative regulation of translation	Any process that stops, prevents, or reduces the frequency, rate or extent of the chemical reactions and pathways resulting in the formation of proteins by the translation of mRNA.
	GO:0035264	multicellular organism growth	The increase in size or mass of an entire multicellular organism, as opposed to cell growth.
	GO:0042035	regulation of cytokine biosynthetic process	Any process that modulates the frequency, rate or extent of the chemical reactions and pathways resulting in the formation of cytokines.
	GO:0042089	cytokine biosynthetic process	The chemical reactions and pathways resulting in the formation of cytokines, any of a group of proteins that function to control the survival, growth and differentiation of tissues and cells, and which have autocrine and paracrine activity.
	GO:0042107	cytokine metabolic process	The chemical reactions and pathways involving cytokines, any of a group of proteins or glycoproteins that function to control the survival, growth and differentiation of tissues and cells, and which have autocrine and paracrine activity.
	GO:0006399	tRNA metabolic process	The chemical reactions and pathways involving tRNA, transfer RNA, a class of relatively small RNA molecules responsible for mediating the insertion of amino acids into the sequence of nascent polypeptide chains during protein synthesis. Transfer RNA is characterized by the presence of many unusual minor bases, the function of which has not been completely established.
	GO:0014065	phosphatidylinositol 3-kinase cascade	A series of reactions, mediated by the intracellular phosphatidylinositol 3-kinase (PI3K). PI3K cascades lie downstream of many cell surface receptor linked signaling pathways and regulate numerous cellular functions.
	GO:0014066	regulation of phosphatidylinositol 3-kinase cascade	Any process that modulates the frequency, rate or extent of signal transduction mediated by the phosphatidylinositol 3-kinase cascade.
	GO:0014068	positive regulation of phosphatidylinositol 3-kinase cascade	Any process that activates or increases the frequency, rate or extent of signal transduction mediated by the phosphatidylinositol 3-kinase cascade.
Turquoise	GO:0006366	transcription from RNA polymerase II promoter	The synthesis of RNA from a DNA template by RNA polymerase II, originating at an RNA polymerase II promoter. Includes transcription of messenger RNA (mRNA) and certain small nuclear RNAs (snRNAs).
	GO:0000003	reproduction	The production by an organism of new individuals that contain some portion of their genetic material inherited from that organism.
	GO:0045596	negative regulation of cell differentiation	Any process that stops, prevents, or reduces the frequency, rate or extent of cell differentiation.
	GO:0006357	regulation of transcription from RNA polymerase II promoter	Any process that modulates the frequency, rate or extent of transcription from an RNA polymerase II promoter.
	GO:0022414	reproductive process	A biological process that directly contributes to the process of producing new individuals by one or two organisms. The new individuals inherit some proportion of their genetic material from the parent or parents.
	GO:0006979	response to oxidative stress	Any process that results in a change in state or activity of a cell or an organism (in terms of movement, secretion, enzyme production, gene expression, etc.) as a result of oxidative stress, a state often resulting from exposure to high levels of reactive oxygen species, e.g. superoxide anions, hydrogen peroxide (H2O2), and hydroxyl radicals.
	GO:0032990	cell part morphogenesis	The process in which the anatomical structures of a cell part are generated and organized.
	GO:0006351	transcription, DNA dependent	The cellular synthesis of RNA on a template of DNA.
Yellow	GO:0045859	regulation of protein kinase activity	Any process that modulates the frequency, rate or extent of protein kinase activity.
	GO:0043549	regulation of kinase activity	Any process that modulates the frequency, rate or extent of kinase activity, the catalysis of the transfer of a phosphate group, usually from ATP, to a substrate molecule.
	GO:0043409	negative regulation of MAPK cascade	Any process that stops, prevents, or reduces the frequency, rate or extent of signal transduction mediated by the MAPKKK cascade.
	GO:0002764	immune response-regulating signaling pathway	The cascade of processes by which a signal interacts with a receptor, causing a change in the level or activity of a second messenger or other downstream target, and ultimately leading to the activation, perpetuation, or inhibition of an immune response.
	GO:0050865	regulation of cell activation	Any process that modulates the frequency, rate or extent of cell activation, the change in the morphology or behavior of a cell resulting from exposure to an activating factor such as a cellular or soluble ligand.
	GO:0002682	regulation of immune system process	Any process that modulates the frequency, rate, or extent of an immune system process.
	GO:0002684	positive regulation of immune system process	Any process that activates or increases the frequency, rate, or extent of an immune system process.
	GO:0010741	negative regulation of intracellular protein kinase cascade	Any process that decreases the rate, frequency or extent of a series of reactions, mediated by protein kinases, which occurs as a result of a single trigger reaction or compound.
	GO:0001932	regulation of protein phosphorylation	Any process that modulates the frequency, rate or extent of addition of phosphate groups into an amino acid in a protein.
	GO:0010627	regulation of intracellular protein kinase cascade	Any process that modulates the rate, frequency or extent of a series of reactions, mediated by protein kinases, which occurs as a result of a single trigger reaction or compound.

Although showing a moderate Z-summary, using the median rank value based on the observed values (less dependent on modules sizes), the turquoise module was only marginally preserved (2 < Z-summary <10). Since this module includes negative regulation of cell differentiation and response to oxidative stress processes, identified deregulated in these same cells [24], Pearson correlation of paired genes were plotted (Fig. 1C). We can observe a change of correlations in SZP compared to CTS (change of color and thickness of lines linking two genes). Whilst co-expressed genes related to regulation of cell cycle processes, protein phosphatase binding and nonsense mRNA decay were extremely conserved between SZP and CTS, genes related to response to negative regulation of cell differentiation and oxidative stress were not.

Next, we defined the hub genes of the modules as those genes with high module connectivity (kME >0.75). This resulted in 13 hubs (*CAPRIN1*, *CLIP4, CTNND1, GRB10, KHDRBS1, KPNA4, LYPD6, NRL, PSTK, RANBP2, RINT1, WASF3* and *ZNF99*) in the highly preserved blue module of CTS, all conserved in SZP (which gained additional four hubs: *ZMIZ1, ORMDL1, EIF5* and *FAM133B*). It is interesting to note that in the yellow and brown modules, the number of hubs in SZP is smaller than in CTS, but all hubs identified in SZP were conserved from CTS, with only one exception in the yellow module (*AKT*) and one in the brown module (*ZMPSTE24*).

The marginally preserved turquoise module had 13 and 16 hub genes in CTS and SZP, respectively, with an overlap of only eight hubs, from which three were previously associated with schizophrenia: *ADARB1* [46], *PNOC* [47] and *XK* [48]. Furthermore, three out of seven hubs found only in SZP were previously associated with schizophrenia. *ADRA2A* is required for normal pre-synaptic control of transmitter release in noradrenergic neurons and has polymorphisms associated to the increased risk of developing the disease [49]. *CIT* is a serine/threonine protein kinase involved in central nervous system development and cancer cells proliferation. Polymorphisms in *CIT* also seems to have an impact in the risk of developing schizophrenia [50]. *LIN7A* encodes a protein involved with the synaptic function and neuron migration in cerebral cortex development [51] and was associated to autism and attention deficit hyperactivity disorder [52, 53]. Even though the other four hubs (*ARMC8, BCL11A, CDO1* and *FOXG1*) have not been linked to schizophrenia, they are candidates for further studies.

## Discussion

In the present study, we characterized molecular alterations during the differentiation of hiPSCs to NPCs from a patient with schizophrenia and health control and analyzed the identified genes in the adult brain tissue from schizophrenia cases and controls to study biological processes that could be disrupted in the disease. As we faced the limitation of a direct comparison due to the different nature of samples (single cell type–neuron–versus all cells types–frontal cortex), we decided to make the analyses using gene co-expression network complemented with the exploration of biological processes. This allowed us to explore genes expressed during neuronal differentiation and important for the development of schizophrenia in the context of adult brain bulk.

It is known that cultured cells are more likely to have genomic instability, genetic and epigenetic mutations [54, 55]. However, we only found one alteration by aCGH, a 1.2 MB gain in SCZP-hiPSC that was maintained in the NPC, suggesting that genes in this region may not be essential for the initial stages of neuron differentiation. Even though none of the 228 DEG was located in this region, it might contains regulatory variants with some role in the disease, although they were not explored in this study. The differentially methylated sites found during neuronal differentiation were preferentially associated with chromatin remodeling complex biological function, an interesting finding considering the previous study where the oxidative stress present in SCZP-NPC was reverted to NPC control levels after treatment of valproic acid (VPA), a histone deacetylase (HDAC) inhibitor [24].

Despite the fact that total RNA was extracted from three independent cell cultures (hiPSC and NPC), we are aware of the limitation of comparing one case with one control, which is the reason why we used the HT-self model for gene expression analyses. This consists in a mathematical model developed to analyze low numbers of samples [28]. Moreover, we used very stringent criteria in all analyses to identify the DEG and overrepresented ontologies (e.g. to be considered overrepresented, each class should contain more than 10 genes and a significant *p*-value after correction for multiple comparisons). Yet, data interpretations present certain limitations.

Concerning the genes involved with neuronal differentiation (hiPSC versus NPC), our comparisons revealed that cells derived from health control displayed twice more DEG than cells derived from the patient with schizophrenia. This finding may be involved with the disruption of cell differentiation that has been described by other studies in schizophrenia. The 228 gene set associated with neuronal differentiation in a patient with schizophrenia had over-representation of pathways classically related to cancer, such as MAPK and metabolic cellular pathways. Whilst new categories may appear with the advance of research in the field, many aspects of normal development and cancer have association to neuronal disorders [56]. There are no implications for tumorigenesis risk, although patients with schizophrenia are at a reduced risk of developing cancer [57]. In our previous study with these cells, we demonstrated that SZCP-NPC had altered metabolism compared to CON-NPC [24]. Likewise, the 228 set had over-representation of genes related to energy, lipid and carbohydrate metabolism, which are altered in cancer [58] and stem cells [59] adding evidence for the implication of tumor-suppressor and oncogenes to neurodevelopment and associated disorders.

Additionally, gene expression profiling from post-mortem brain tissue of patients with schizophrenia and controls were studied. To begin with, although there were no significant differences in age or PMI, the pH was slightly lower in the brain of patients with schizophrenia, a characteristic that has been described although not considered for the analyses [15]. The pH may have an effect on the expression of some genes or these genes might be regulating the pH change. We found that 78 out of 233 DEG identified by the comparison between brain bulks from patients with schizophrenia and health controls had their expression also associated to differences in pH, an issue that needs to be deeper investigated.

Likewise, to explore the relationship between genes involved in neuronal differentiation and fully differentiated brains in patients with schizophrenia, we have to consider some additional known limitations: (1) Use of medication, hypoxia and/or injury are known confounding factors in the samples derived from the patients; (2) Comparison between data generated by single cell types (hiPSC and NPC) with data representing a mixed cell population at the end stage of the disease (a “whole brain tissue” that includes the glia, immunological and vascular cells); 3) Different methods were used to identify differentially expressed genes; (4) Genes from neurodevelopment are not being expressed or are being expressed at very low levels once the brain has ended its maturation. Supporting this later hypothesis, the comparison of the 228 and 233 DEG with modules derived from developing brains [31], showed that even though both sets have genes associated with neurodevelopment, they are representative of different stages. Thus, the overlap of only four genes was not a surprise.

Consequently, we decided to study the 228 gene set using co-expression modules in the expression data from the cohort of post-mortem cases of schizophrenia and controls identifying a well preserved module (blue) and, although not completely, a less conserved module (turquoise). The blue module was associated with regulation of cell cycle progress, protein phosphatase binding and nonsense mRNA decay. Genes from this module have function related to genome maintenance, RNA integrity, cell proliferation and/or differentiation, such as *ANPC4*, *SPHK1* and *SPP* [60–62].

The turquoise module displays a set of genes involved in negative regulation of cell differentiation and oxidative stress response, more specifically reactive oxygen species (ROS) production, mitochondria function and neuron differentiation, which are closely related to the functional study of these cells that exhibited deregulation of mitochondria function in SZCP-NPC [24]. Among the genes represented in the module, three genes have a described function associated with ROS generation. *ASK1* is a member of the mitogen-activated protein kinase family activated by cellular stress, activating c-jun N-terminal kinase (JNK) and p38 in response to oxidative stress, endoplasmic reticulum stress, infection and calcium influx [63]. *PNPT1* is predominantly localized in the mitochondrial inter-membrane space and is implicated in RNA targeting to human mitochondria, small noncoding nuclear RNAs (5S rRNA, MRP RNA, some tRNAs, and miRNAs) [64]. cAMP-induced cFos is a transcriptional repressor of *CD44* that triggers ROS generation [65]. Additionally, some hub genes found exclusively in SZP have been associated to neuronal plasticity and different cancers, most of them associated to cell proliferation. Likewise, some of the hub genes have already been associated to neurodevelopment disorders.

Mitochondria activity and energy metabolism undergo dramatic remodeling during embryonic development. Early embryos are initially dependent upon oxidative metabolism due to inheritance of maternal mitochondria which are subsequently segregated among daughter cells. A predominantly glycolytic metabolism provides sufficient energy to support stem cells in basal state, but a robust metabolic network is required to adequate the increasingly energetic demands [66]. The established initial mechanism support flux through the tricarboxylic acid cycle and electron transport chain whilst mitochondrial oxidization of pyruvate and glutamine to CO_2_ is optimized to extract maximal energetic supply. It has been suggested that a concomitant rise in mitochondrial reactive oxygen species may prime stem cells for lineage differentiation [66, 67]. The large number of observations identifying stem cell properties affected by energy-responsive molecules and signaling pathways raises questions about the fate of stem cells under conditions when metabolic homeostasis is perturbed. The literature suggests that abnormal endocrine signaling in organisms in extreme metabolic states has a substantial impact on proliferation and differentiation of multiple stem cell populations throughout the body [68]. It is also possible that variations in metabolism during gestation contribute to changes in offspring through their effects on stem cells. For example, low energy levels and the associated hormonal signals that occur in the pregnant mother could be directly transmitted to the offspring through the placenta, resulting in either transient or permanent changes in embryonic stem cells. Therefore, it seems that risk factors for schizophrenia such as famine, hipoxia and low birth weight could be implicated in permanent changes to stem cells leading to altered brain neural-development, as well as some alterations that are still presented in adulthoods, like disturbed neural plasticity and increased ROS production.

## Conclusions

In this study, data integration provided by different sample types (single cell versus tissue bulk) and methodologies were used to contribute to a better understating the schizophrenia. This model allowed us to examine the influence of the neuronal cells to the schizophrenic brain through the involvement of genes related to neuronal differentiation and cell metabolism. We identified a non-conserved module based on a co-expression network in schizophrenia compared to health controls that enabled us to predict that metabolic changes involving oxidative stress and negative regulation of cell differentiation may be occurring during early neurodevelopment and prompting to the development if schizophrenia. Several genes from this module were associated to schizophrenia by other studies and we revealed new candidates (*ARMC8, BCL11A, CDO1* and *FOXG1*) to be disrupted during neuronal differentiation in patients with schizophrenia.

## Additional file

Additional file 1: **This is a PDF document containing tables (1 to 5), images (1 to 4) with respective legends that exhibit selected findings from the research that were deemed less important to the overall message than the figure and tables included in the main body of this manuscript.**

## Abbreviations

hiPSC: Human induced pluripotent cells
SCZP: Schizophrenia
CON: Control individual
NPCs: Neural progenitor cells
ROS: Reactive oxygen species
CNVs: Copy number variations
CGH: Comparative genomic hybridization
CTS: Controls subjects
SZP: Patients with schizophrenia
ROS: Reactive oxygen species
SAM: Significant Analysis of Microarray
WGCNA: Weighted Gene Co-expression Network Analysis
TOM: Topological overlap measure
adjP: Adjusted *p*-value
FDR: False discovery rate
PC: Pearson Correlation
