# Supplementary material for: Co-expression network of neural-differentiation genes shows specific pattern in schizophrenia
Source: BMC Med Genomics. 2015 May 16;8:23. doi: 10.1186/s12920-015-0098-9 (PMC4493810; doi:10.1186/s12920-015-0098-9)
Supplement: Supplementary file 1 — This is a PDF document containing tables (1 to 5), images (1 to 4) with respective legends that exhibit selected findings from the research that were deemed less important to the overall message than the figure and tables included in the main body of this manuscript. [file 12920_2015_98_MOESM1_ESM.pdf]

Supplemental Figure 1 – Gain of 1.2 MB at 20q11.21 acquired 1by hiPSC and maintained in the NPC. The region contains 32 genes.

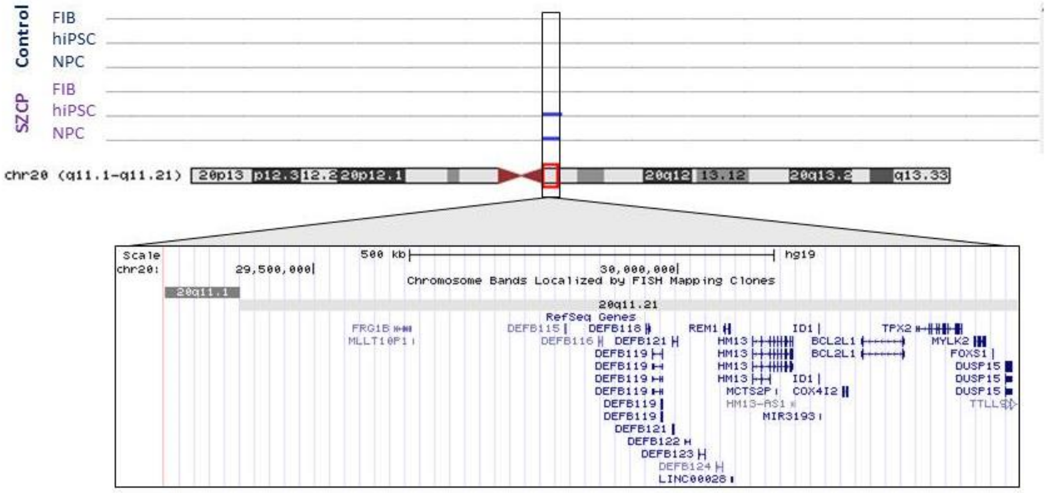

Supplemental Figure 2 –Venn-diagrams constructed to select CpGs associated with neural-differentiation in a SCZP. Selected CpGs are highlighted in red. Selection of (**A)** hypo- and (**B)** hyper-methylated CpGs in control; (**C**) hypo- and (**D**) hyper-methylated CpGs in SCZP. CpGs that were exclusively (**E**) hypo-methylated or (**F**) hyper-methylated in SCZP.

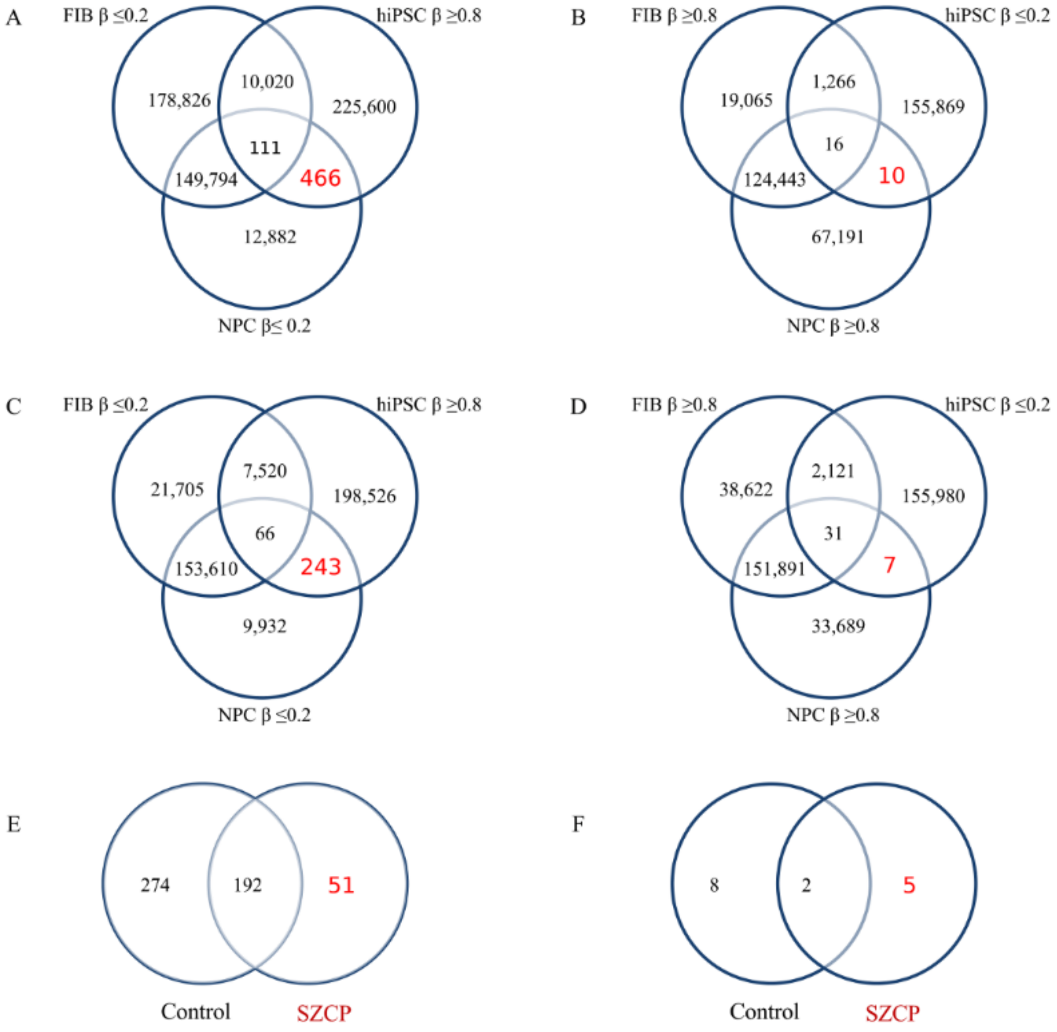

Supplemental Figure 3 – Diagram containing the different analysis steps with gene expression data.

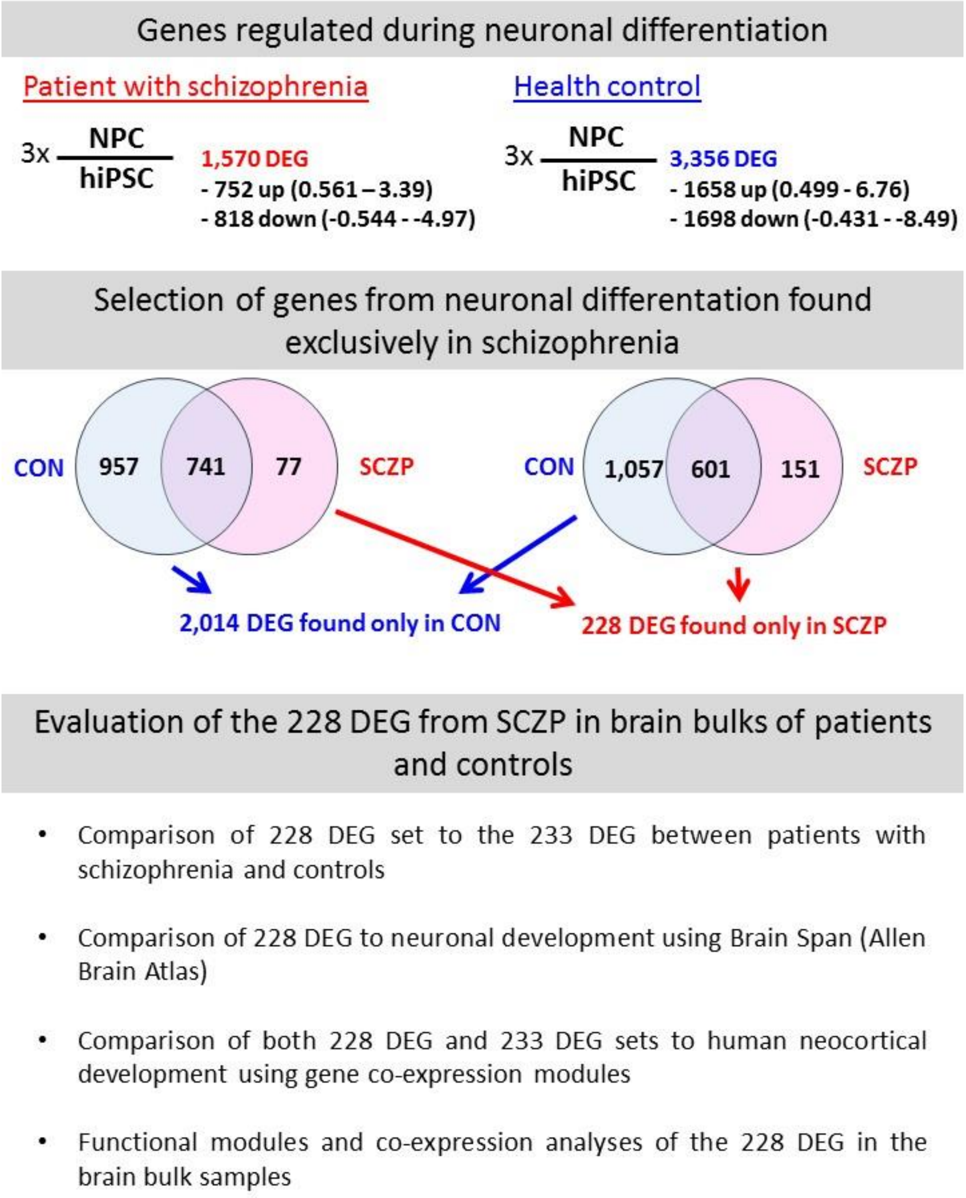

Supplemental Figure 4 - Scatter smooth of module eigengene values identified by Parikshak et al (2013) at different stages of neocortical development. The module eigengene values are defined as the first principal component (PC) of the expression matrix. Modules eigengene values are represented in y-axis and age are provided in days. Different periods of lifetime are scaled from light to dark blue colors: early foetal, early-mid foetal, late mid-foetal, late foetal, neonatal/early infancy and late infancy. The red line indicates the birth. Graphs show the distribution of the 228 DEG (in M8) and 233 DEG (in M1, M5 and M15) only in the modules that were statistically significant enriched to one of our datasets. M8 comprises genes preferentially expressed in early foetal period; M1, M5 and M15 comprise genes preferentially expressed in mid-foetal or late foetal and infancy periods.

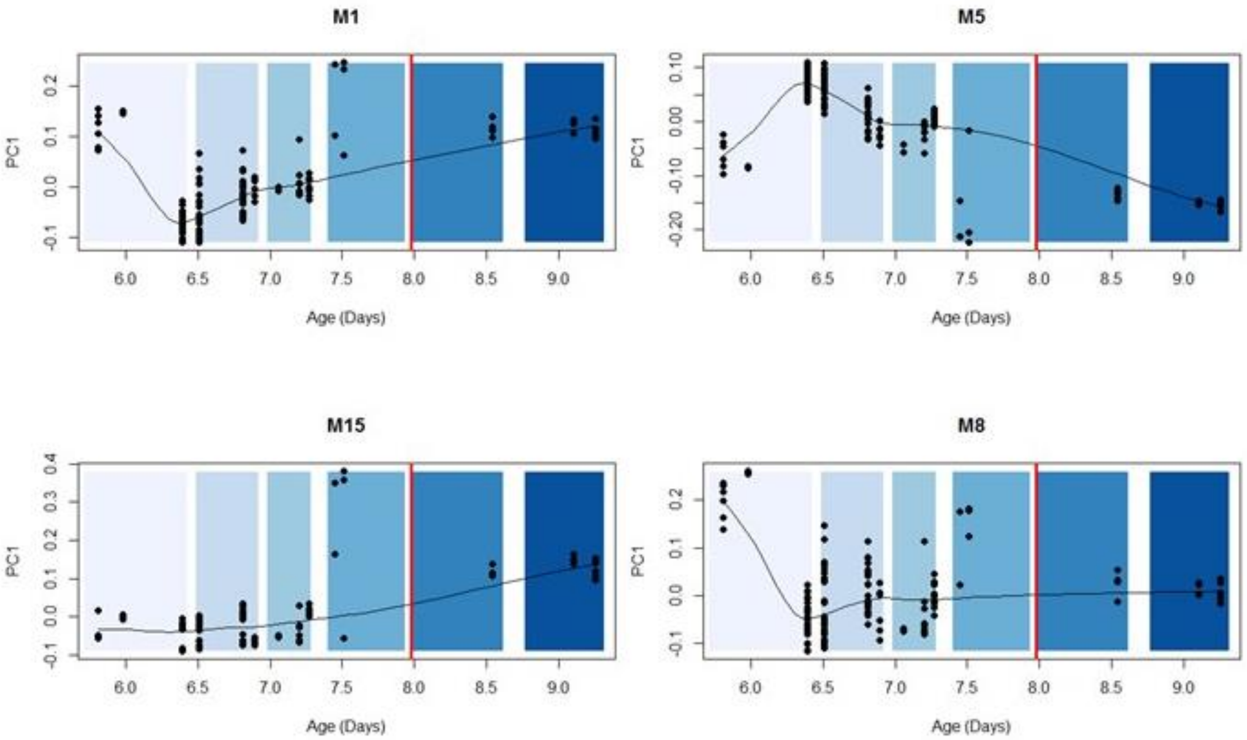

Supplemental Table 1 – Genes found hypo- and hyper-methylated in SCZP.

**Hypo-methylated genes**				**Hyper-methylated genes**			
**Gene**	**Chr**	***Mapinfo***	**Relation to UCSC CpG island**	**Gene**	**Chr**	***Mapinfo***	**Relation to UCSC CpG island**
ERRFI1	1	8086959	S_Shore	*HK1*	10	71149910	
RERE	1	8509372		*LOC145845*	15	37170816	N_Shore
ZBTB40	1	22782452	S_Shelf	CYP2F1	19	41632920	N_Shore
GULP1	2	189382445					
OBSL1	2	220436936	S_Shore				
SH3BP4	2	235907796					
VGLL4	3	11651881					
PLSCR5	3	146323078					
PLCH1	3	155394303					
SORBS2	4	186732926					
SEPT8	5	132111426	N_Shore				
SLC25A13	7	95811907					
SRPK2	7	104945248	S_Shelf				
PRRT4	7	127997529	N_Shelf				
SMARCA2	9	2044055	N_Shelf				
PALM2	9	112593847					
MPP7	10	28436499					
FUT11	10	75533431	S_Shore				
NAV2	11	19546133					
C11orf9	11	61541027	N_Shelf				
EXPH5	11	108409825					
BACE1	11	117171073					
UBASH3B	11	122655638					
TMCC3	12	95010113					
FGD6	12	95477853					
FREM2	13	39358795					
FARP1	13	98919378					
DPF3	14	73209128					
TTLL5	14	76309157					
ATG2B	14	96752011					
MEOX1	17	41739246					
LRRN4	20	6035902	S_Shelf				
ARSF	X	2984985					

Supplemental Table 2 – Set of 228 genes found altered during neural-differentiation of the SCZP cells.

up-regulated genes

ACTA1	1q42.13	*FH*	1q42.1	*NRL*	14q11.1-q11.2	*WASF3*	13q12
ADRA2A	10q25.2	*FLT1*	13q12	*NUP107*	12q15	*WASL*	7q31.3
ADRBK2	22q12.1	*FOXG1*	14q13	*ORMDL1*	2q32	*WDR44*	Xq24
AGL	1p21	*FOXN2*	2p22-p16	*PC*	11q13.4-q13.5	*WIF1*	12q14.3
ANAPC4	4p15.2	*FZD5*	2q33.3	*PDE4B*	1p31	*XIST*	Xq13.2
ARMC8	3q22.3	*GAL3ST1*	22q12.2	*PIK3C2A*	11p15.5-p14	*XK*	Xp21.1
ASNS	7q21.3	*GFOD1*	6pter-p22.1	*PMAIP1*	18q21.32	*YWHAG*	7q11.23
ASPM	1q31	*GIGYF2*	2q37.1	*PNMA1*	14q24.3	*ZGLP1*	19p13.2
ATF4	22q13.1	*GJC1*	17q21.31	*PNMT*	17q	*ZMPSTE24*	1p34
ATP6V1C1	8q22.3	*GMFB*	14q22.2	*PNPT1*	2p15	*ZNF273*	7q11.21
BCLAF1	6q22-q23	*GRB10*	7p12.2	*PPM1B*	2p21	*ZNF426*	19p13.2
BID	22q11.1	*GTF2H1*	11p15.1-p14	*PRDX4*	Xp22.11	*ZNF614*	19q13.41
FAM204A	10q26.11	*HDAC2*	6q21	*PSMB10*	16q22.1	*ZNF99*	19p12
CACNA1E	1q25-q31	*HEY2*	6q21	*PSMD7*	16q22.3		
CACNA1H	16p13.3	*HISPPD1*	5q21.1	*PSTK*	10q26.13		
CADM3	1q21.2-q22	*HLA-DOA*	6p21.3	*PTPN12*	7q11.23		
CAPRIN1	11p13	*HNRNPH1*	5q35.3	*PYGL*	14q21-q22		
CASC5	15q14	*HOXB9*	17q21.3	*RAD54B*	8q22.1		
CCAR1	10q21.3	*HRK*	12q24.22	*RANBP2*	2q12.3		
CD24	6q21	*HUWE1*	Xp11.22	*RANGRF*	17p13.1		
CDK5R1	17q11.2	*IMPA1*	8q21.13-q21.3	*RARS*	5q35.1		
CDO1	5q23.2	*ING3*	7q31	*RB1CC1*	8q11		
CENPJ	13q12.12	*ING5*	2q37.3	*RBM16*	6q25.1-q25.3		
CHUK	10q24-q25	*JAZF1*	7p15.2-p15.1	*RCBTB1*	13q14		
CIT	12q24	*KHDRBS1*	1p32	*RINT1*	7q22.3		
COL9A3	20q13.3	*KPNA4*	3q25.33	*SARNP*	12q13.2		
CPOX	3q12	*LIN7A*	12q21	*SFRS1*	17q22		
CTSF	11q13	*LIPA*	10q23.2-q23.3	*SGK1*	6q23		
CXCL5	4q13.3	*LOC389634*	12p13.31	*SLC38A4*	12q13		
DCLRE1A	10q25.1	*LOC645937*	9q22.1	*SMG5*	1q21.2		
DDX6	11q23.3	*LRRC49*	15q23	*SMO*	7q32.3		
DHX36	3p13-q23	*METAP2*	12q22	*SNORD3B-1*	17p11.2		
DOK1	2p13	*METT10D*	17p13.3	*SNTB1*	8q23-q24		
DPM1	20q13.13	*MICB*	6p21.3	*SOX3*	Xq27.1		

DUSP6	12q22-q23	*MIF*	22q11.23	*SPRY4*	5q31.3
DYNC2H1	11q21-q22.1	*MRPL19*	2p11.1-q11.2	*SSTR1*	14q13
E2F3	6p22	*MTF1*	1p33	*STX4*	16p11.2
EED	11q14.2-q22.3	*MTHFS*	15q25.1	*SYPL2*	1p13.3
EIF3A	10q26	*MYB*	6q22-q23	*SYT14*	1q32.2
EIF3B	7p22.3	*MYBL1*	8q22	*TBK1*	12q14.1
EIF5	14q32.32	*NEFH*	22q12.2	*TEK*	9p21
EPC1	10p11	*NKRF*	Xq24	*TMEM208*	16q22.1
ETV1	7p21.3	*NLGN1*	3q26.31	*TOP3A*	17p12-p11.2
FAM133B	7q21.2	*NLK*	17q11.2	*TRDMT1*	10p15.1
FEZ1	11q24.2	*NOC3L*	10q23.33	*TRIM24*	7q32-q34
FGD4	12p11.21	*NPR1*	1q21-q22	*USP1*	1p31.3
down-regulated genes					
ACCN1	17q11.2-q12	*MAP3K5*	6q22.33		
ADARB1	21q22.3	*MDGA1*	6p21		
ADIPOR2	12p13.31	*MGAT4B*	5q35		
AKT1	14q32.32	*MOCS3*	20q13.13		
ALDH1A1	9q21.13	*NDEL1*	17p13.1		
APOA1	11q23-q24	*NEK6*	9q33.3-q34.11		
APOA2	1q21-q23	*NTF3*	12p13		
AQP1	7p14	*PACS1*	11q13.1-q13.2		
ARMCX6	Xq21.33-q22.3	*PDGFA*	7p22		
ATXN7	3p21.1-p12	*PDK2*	17q21.33		
BACE1	11q23.2-q23.3	*PEX6*	6p21.1		
BCL11A	2p16.1	*PFN2*	3q25.1		
CACNA1C	12p13.3	*PLOD2*	3q24		
CCDC149	4p15.2	*PNOC*	8p21		
CEACAM1	19q13.2	*PPAP2B*	1p32.2		
CEBPB	20q13.1	*PPIB*	15q21-q22		
CLDN11	3q26.2-q26.3	*PPP1R13B*	14q32.33		
CLIP4	2p23.2	*PRDM16*	1p36.23-p33		
COL16A1	1p35-p34	*RABEP2*	16p11.2		
CPE	4q32.3	*RARA*	17q21		
CST3	20p11.21	*RBM20*	10q25.2		
CTDSP1	2q35	*RHOBTB2*	8p21.3		

CTNND1	11q11	*RIPK4*	21q22.3
CUX1	7q22.1	*RPL11*	1p36.1-p35
DCT	13q32	*RPLP2*	11p15.5
DUX4	10q26.3	*S100A7*	1q21
EDNRA	4q31.22	*SPHK1*	17q25.2
EPHA2	1p36	*SPI1*	11p11.2
FBRS	16p11.2	*SSC5D*	19q13.42
FOS	14q24.3	*TICAM1*	19p13.3
FURIN	15q26.1	*USH1G*	17q25.1
FXYD3	19q13.12	*ZMIZ1*	10q22.3
GPR155	2q31.1		
HAAO	2p21		
HS6ST2	Xq26.2		
HSD17B8	6p21.3		
HTRA1	10q26.3		
IL13RA1	Xq24		
ITGA2	5q11.2		
KCNK15	20q13.12		
KDELC2	11q22.3		
LGALS3	14q22.3		
LOXL1	15q22		
LYPD6	2q23.2		
MANEA	6q16.1		

Supplemental Table 3 – Genes from the 228 gene set that have been described in schizophrenia studies

Gene	Information retrieved from Gene/NCBI (Summary)
NDEL1	This gene encodes a coiled-coil protein that plays a role in multiple processes including cytoskeletal organization, cell signaling and neuron migration, outgrowth and maintenance.
ATF4	This gene encodes a transcription factor that was originally identified as a widely expressed mammalian DNA binding protein that could bind a tax-responsive enhancer element in the LTR of HTLV-1. The encoded protein was also isolated and characterized as the cAMP-response element binding protein 2 (CREB-2). The protein encoded by this gene belongs to a family of DNA-binding proteins that includes the AP-1 family of transcription factors, cAMP-response element binding proteins (CREBs) and CREB-like proteins. These transcription factors share a leucine zipper region that is involved in protein-protein interactions, located C-terminal to a stretch of basic amino acids that functions as a DNA binding domain. Two alternative transcripts encoding the same protein have been described. Two pseudogenes are located on the X chromosome at q28 in a region containing a large inverted duplication.
AKT1	The serine-threonine protein kinase encoded by the AKT1 gene is catalytically inactive in serum-starved primary and immortalized fibroblasts. AKT1 and the related AKT2 are activated by platelet-derived growth factor. The activation is rapid and specific, and it is abrogated by mutations in the pleckstrin homology domain of AKT1. It was shown that the activation occurs through phosphatidylinositol 3-kinase. In the developing nervous system AKT is a critical mediator of growth factor-induced neuronal survival. Survival factors can suppress apoptosis in a transcription-independent manner by activating the serine/threonine kinase AKT1, which then phosphorylates and inactivates components of the apoptotic machinery. Mutations in this gene have been associated with the Proteus syndrome. Multiple alternatively spliced transcript variants have been found for this gene
MDGA1	Not available
PDE4B	This gene is a member of the type IV, cyclic AMP (cAMP)-specific, cyclic nucleotide phosphodiesterase (PDE) family. Cyclic nucleotides are important second messengers that regulate and mediate a number of cellular responses to extracellular signals, such as hormones, light, and neurotransmitters. The cyclic nucleotide phosphodiesterases (PDEs) regulate the cellular concentrations of cyclic nucleotides and thereby play a role in signal transduction. This gene encodes a protein that specifically hydrolyzes cAMP. Altered activity of this protein has been associated with schizophrenia and bipolar affective disorder.
FEZ1	This gene is an ortholog of the C. elegans unc-76 gene, which is necessary for normal axonal bundling and elongation within axon bundles. Expression of this gene in C. elegans unc-76 mutants can restore to the mutants partial locomotion and axonal fasciculation, suggesting that it also functions in axonal outgrowth. The N-terminal half of the gene product is highly acidic.
TF3	The protein encoded by this gene is a member of the neurotrophin family, that controls survival and differentiation of mammalian neurons. This protein is closely related to both nerve growth factor and brain-derived neurotrophic factor. It may be involved in the maintenance of the adult nervous system, and may affect development of neurons in the embryo when it is expressed in human placenta. NTF3-deficient mice generated by gene targeting display severe movement defects of the limbs.
ADRBK2	The beta-adrenergic receptor kinase specifically phosphorylates the agonist-occupied form of the beta-adrenergic and related G protein-coupled receptors. Overall, the beta adrenergic receptor kinase 2 has 85% amino acid similarity with beta adrenergic receptor kinase 1, with the protein kinase catalytic domain having 95% similarity. These data suggest the existence of a family of receptor kinases which may serve broadly to regulate receptor function.
CACNA1C	This gene encodes an alpha-1 subunit of a voltage-dependent calcium channel. Calcium channels mediate the influx of calcium ions into the cell upon membrane polarization. The alpha-1 subunit consists of 24 transmembrane segments and forms the pore through which ions pass into the cell. The calcium channel consists of a complex of alpha-1, alpha-2/delta, beta, and gamma subunits in a 1:1:1:1 ratio. There are multiple isoforms of each of these proteins, either encoded by different genes or the result of alternative splicing of transcripts. The protein encoded by this gene binds to and is inhibited by dihydropyridine.

ADRA2A	Alpha-2-adrenergic receptors are members of the G protein-coupled receptor superfamily. They include 3 highly homologous subtypes: alpha2A, alpha2B, and alpha2C. These receptors have a critical role in regulating neurotransmitter release from sympathetic nerves and from adrenergic neurons in the central nervous system. Studies in mouse revealed that both the alpha2A and alpha2C subtypes were required for normal presynaptic control of transmitter release from sympathetic nerves in the heart and from central noradrenergic neurons; the alpha2A subtype inhibited transmitter release at high stimulation frequencies, whereas the alpha2C subtype modulated neurotransmission at lower levels of nerve activity. This gene encodes alpha2A subtype and it contains no introns in either its coding or untranslated sequences

Supplemental Table 4 – Transcripts from the 233 DEG that were affected by pH.

Transcripts	ENTREZID	GENE NAME
A_24_P715434	NA	NA
A_32_P204565	NA	NA
A_32_P218131	NA	NA
A_32_P58912	NA	NA
AK024584	NA	NA
AK098638	NA	NA
AK123157	NA	NA
ALDH1L1	10840	aldehyde dehydrogenase 1 family, member L1
ARHGEF10	9639	Rho guanine nucleotide exchange factor (GEF) 10
ATP13A4	84239	ATPase type 13A4
ATP1B2	482	ATPase, Na+/K+ transporting, beta 2 polypeptide
BBS2	583	Bardet-Biedl syndrome 2
BC042172	NA	NA
BC062473	NA	NA
C1R	715	complement component 1, r subcomponent
GLIS3-AS1	84850	GLIS3 antisense RNA 1
CD52	1043	CD52 molecule
CR626626	NA	NA
DCAKD	79877	dephospho-CoA kinase domain containing
DPYSL2	1808	dihydropyrimidinase-like 2
DUSP1	1843	dual specificity phosphatase 1
DUSP2	1844	dual specificity phosphatase 2
DYNLL2	140735	dynein, light chain, LC8-type 2
ECHDC2	55268	enoyl CoA hydratase domain containing 2
EGFR	1956	epidermal growth factor receptor
EGR2	1959	early growth response 2
SLC14A1	6563	solute carrier family 14 (urea transporter), member 1 (Kidd blood group)
POU2F1	5451	POU class 2 homeobox 1
ENST00000379884	NA	NA
FKBP5	2289	FK506 binding protein 5
FLJ38359	NA	NA
GAA	2548	glucosidase, alpha; acid
GOT2	2806	glutamic-oxaloacetic transaminase 2, mitochondrial
HIF3A	64344	hypoxia inducible factor 3, alpha subunit
HSPBP1	23640	HSPA (heat shock 70kDa) binding protein, cytoplasmic cochaperone 1
IFT88	8100	intraflagellar transport 88 homolog (Chlamydomonas)
TECPR2	9895	tectonin beta-propeller repeat containing 2
LOC441244	NA	NA
LOC92154	NA	NA
LPAAT-THETA	NA	NA
M69296	NA	NA
CDKN2AIPNL	91368	CDKN2A interacting protein N-terminal like
MGC42105	NA	NA

MRVI1	10335	murine retrovirus integration site 1 homolog
MT1X	4501	metallothionein 1X
SND1-IT1	27099	SND1 intronic transcript 1 (non-protein coding)
NETO2	81831	neuropilin (NRP) and tolloid (TLL)-like 2
NHLRC2	374354	NHL repeat containing 2
NIP7	51388	NIP7, nucleolar pre-rRNA processing protein
PALLD	23022	palladin, cytoskeletal associated protein
PIGA	5277	phosphatidylinositol glycan anchor biosynthesis, class A
FERMT2	10979	fermitin family member 2
PLIN1	5346	perilipin 1
POLR3H	171568	polymerase (RNA) III (DNA directed) polypeptide H (22.9kD)
PPAPDC1B	84513	phosphatidic acid phosphatase type 2 domain containing 1B
RAB34	83871	RAB34, member RAS oncogene family
RHCE	6006	Rh blood group, CcEe antigens
RMND5B	64777	required for meiotic nuclear division 5 homolog B (S. cerevisiae)
SCGB1D1	10648	secretoglobin, family 1D, member 1
SDC4	6385	syndecan 4
SEC31A	22872	SEC31 homolog A (S. cerevisiae)
SLC15A2	6565	solute carrier family 15 (oligopeptide transporter), member 2
SLC29A1	2030	solute carrier family 29 (equilibrative nucleoside transporter), member 1
SOD3	6649	superoxide dismutase 3, extracellular
THC2364621	NA	NA
THC2404308	NA	NA
THC2407386	NA	NA
THC2443552	NA	NA
THC2446669	NA	NA
THC2453189	NA	NA
TMEM38A	79041	transmembrane protein 38A
TMPRSS5	80975	transmembrane protease, serine 5
TPCN1	53373	two pore segment channel 1
TTC18	118491	tetratricopeptide repeat domain 18
TTC7A	57217	tetratricopeptide repeat domain 7A
ZMYM5	9205	zinc finger, MYM-type 5
ZNF442	79973	zinc finger protein 442
ZSCAN25	221785	zinc finger and SCAN domain containing 25

Supplemental Table 5 – Analyses of the 228 DEG genes from the comparison that revealed genes associated to neuronal development related to schizophrenia and the 233 DEG from the comparison between brain bulks of patients with schizophrenia and health control. This analysis compared both set of DEGs to the modules extracted from neocortical development described in Parikshak et al (2013).

Brain Development Modules	Set of 228 DEG (pvalue)	Set of 233 DEG (pvalue)
M1	0,306	**0,038**
M2	0,822	0,656
M3	0,100	0,908
M4	0,650	0,961
M5	0,858	**0,048**
M6	0,188	1,000
M8	**0,015**	0,567
M9	0,142	0,861
M10	1,000	1,000
M11	0,518	0,394
M12	0,988	0,964
M13	0,534	0,306
M14	0,170	1,000
M15	0,224	**0,001**
M16	0,233	0,789
M17	0,169	0,996
M18	0,489	0,241

M8 and M15 were representative of genes preferentially expressed in "early" and "late" foetal period in human brain development, respectively.
